# Development and psychometric evaluation of the Liver Disease Stigma Scale (LDSS)

**DOI:** 10.1016/j.jhepr.2026.101870

**Published:** 2026-05-06

**Authors:** Wei Zhang, Edward Wu, Kelly Hsu, Cassidy Sun, Toshali Katyal, Ana Ivkovic, Heidi Yeh, Emily Bethea, Sarah Wakeman, Russell Goodman, Jay Luther, Cristal Brown, Robert Wong, Esperance Schaefer, Raymond T. Chung, Nneka Ufere, Annie B. Fox

**Affiliations:** 1 Liver Center, Gastroenterology Division, Massachusetts General Hospital, Boston, MA, USA; 2 Tufts University School of Medicine, Boston, MA, USA; 3 Harvard T.H. Chan School of Public Health, Cambridge, MA, USA; 4 Department of Psychiatry, Massachusetts General Hospital, Boston, MA, USA; 5 Liver Transplant Center, Massachusetts General Hospital, Boston, MA, USA; 6 Department of Medicine, Massachusetts General Hospital, Boston, MA, USA; 7 Division of Gastroenterology, Dell Medical School at UT Austin, Austin, TX, USA; 8 Division of Gastroenterology, Stanford University, Stanford, CA, USA; 9 Division of Gastroenterology, Brigham and Women’s Hospital, Boston, MA, USA; 10 Healthcare Data Analytics, MGH Institute of Health Professions, Boston, MA, USA

**Keywords:** Patient-reported outcome measures, Social stigma, Healthcare disparities, Liver cirrhosis, Self-report, Scale validation, Substance use disorders, Care avoidance

## Abstract

**Background & Aims:**

Chronic liver disease (CLD) is increasingly prevalent, and stigma remains a barrier to care, particularly for alcohol-associated liver disease (ALD). No validated instruments measure liver disease-specific stigma. Thus, we developed and evaluate here the Liver Disease Stigma Scale (LDSS).

**Methods:**

We conducted a cross-sectional study of 211 patients from inpatient and outpatient hepatology services at a tertiary center. Participants were categorized as ALD (n = 128) or non-ALD (n = 83). The LDSS assessed internalized, anticipated (family and healthcare), and experienced (family and healthcare) stigma. Psychometric evaluation included exploratory factor analysis (EFA), internal consistency (Cronbach’s α), and convergent validity with the Substance Use Stigma Mechanisms Scale (SU-SMS) and mental health symptoms. Known-groups validity was assessed using *t* tests and adjusted linear regression.

**Results:**

EFA supported a five-factor structure accounting for 73.7% of variance. Subscale reliability was excellent (α = 0.90–0.97). LDSS subscales showed strong convergent validity with corresponding SU-SMS subscales (r = 0.43–0.80) and moderate correlations with mental health (r = 0.18–0.60). Known-groups validity was demonstrated by higher stigma scores among participants with ALD, particularly for internalized stigma (2.34 *vs.* 1.50, *p* <0.001, d = 0.85) and family-experienced stigma (1.80 *vs.* 1.18, *p* <0.001, d = 0.77). After adjustment, differences in internalized and family-experienced stigma remained significant.

**Conclusions:**

The LDSS demonstrated a stable five-factor structure, strong reliability, and good convergent validity, supporting its use as a psychometrically sound measure of liver disease-specific stigma. Patients with ALD showed higher stigma levels, underscoring the clinical relevance of the scale and the need for further research on stigma and outcomes in liver disease. However, findings require validation in larger, more diverse, and independent samples.

**Impact and implications:**

The LDSS fills an important measurement gap by providing the first multidimensional instrument specifically developed to assess stigma among adults with CLD. Stigma levels were higher among individuals with ALD, underscoring the relevance of stigma as a psychosocial factor that can influence engagement and wellbeing in this population. The scale provides a standardized and psychometrically robust approach for assessing stigma and offers a foundation for future research and quality improvement efforts aimed at identifying stigma burden and informing targeted clinical or behavioral strategies. These findings could help clinicians, researchers, and health systems more systematically understand how stigma shapes patient experience and consider approaches to promote more equitable and person-centered liver disease care.

## Highlights

• This is the first psychometric evaluation of a scale measuring liver disease stigma.
• The scale has a five-factor structure with strong reliability and convergent validity.
• ALD was linked to higher internalized and family stigma.
• The scale provides a standardized tool to assess stigma in liver disease.

## Graphical abstract

**Figure:**
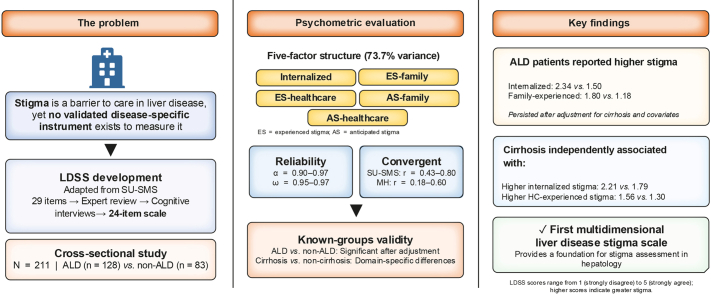

## Introduction

Chronic liver disease (CLD) and cirrhosis represent a growing global health concern, with prevalence rising substantially over the past two decades.^1^ In the United States, this increase has been driven largely by alcohol-associated liver disease (ALD) and metabolic dysfunction-associated steatotic liver disease (MASLD), which have now surpassed viral hepatitis as leading causes of liver-related mortality.[2], [3], [4]

Stigma represents a crucial, yet underexplored barrier to optimal hepatology care, particularly for patients with ALD.^5^^,^^6^ Stigma contributes to delayed diagnosis, reduced adherence, poor engagement in care, and inequities in transplant evaluation and listing.^7^^,^^8^ These effects operate through three interconnected mechanisms: internalized stigma (IS; the application of negative stereotypes and prejudice to the self), anticipated stigma (AS; expectation of judgment or rejection), and experienced stigma (ES: actual discrimination from healthcare workers, family, or society).[9], [10], [11] Each mechanism exerts distinct influences on patient behavior and clinical outcomes. IS is linked to depression and reduced self-worth, AS leads to care avoidance and nondisclosure, and ES fosters mistrust and social withdrawal.[12], [13], [14], [15]

Despite increasing recognition of the impact of stigma in conditions such as HIV, mental illness, and substance use disorders, its role in liver disease remains poorly characterized and inadequately measured.[16], [17], [18] Existing studies document stigma in viral hepatitis and cirrhosis populations, but few examine distinct stigma mechanisms across liver disease etiologies or settings.^8^^,^^19^ Although validated stigma measures exist for other conditions, no instrument has been specifically adapted and validated for liver disease populations. Furthermore, no stigma measure has been psychometrically validated across diverse liver disease etiologies to enable comparisons between conditions such as ALD and non-alcohol-associated liver disease. This gap limits our ability to identify patients experiencing high stigma burden and to evaluate interventions aimed at reducing stigma in clinical hepatology practice.

To address this gap, we developed the Liver Disease Stigma Scale (LDSS) by adapting the validated Substance Use Stigma Mechanisms Scale (SU-SM) for liver disease population.^20^ This adaptation included modifying stereotype-related items from the original scale, such as assumptions about pill-seeking behavior, to reflect attribution-based stereotypes relevant to liver disease, including assumptions about alcohol use. The LDSS is a theory-informed, multidimensional instrument assessing IS, AS, and ES among patients with CLD. We evaluated its psychometric properties, including factor structure, reliability, and convergent validity with related constructs. Finally, we compared stigma levels across disease etiologies, hypothesizing that patients with ALD would report the highest stigma burden because of persistent behavioral attribution and moral judgment surrounding alcohol use.

## Material and methods

### Study design and participants

We conducted a cross-sectional survey of adult patients with CLD at Massachusetts General Hospital (MGH), Boston, MA, USA between October 2023 and August 2024. Patients were recruited from the inpatient hepatology consult service and outpatient specialty hepatology clinics. Inpatient participants were identified through daily census review; outpatient participants were identified through scheduled clinic appointment lists. Eligible patients were approached in person by study staff during their clinical encounter. Inclusion criteria included age ≥18 years and a confirmed diagnosis of CLD. CLD was defined as hepatic disease lasting at least 6 months, confirmed by clinical history, imaging, laboratory findings, elastography, or histology.

CLD etiology was categorized as ALD (alcohol-associated cirrhosis, alcohol-associated fatty liver disease, or alcohol-associated hepatitis), cholestatic liver disease, such as primary biliary cholangitis or primary sclerosing cholangitis, MASLD, viral hepatitis, CLD with multiple etiologies, and other causes of CLD, which included autoimmune hepatitis and less common etiologies. For patients with more than one contributing etiology, ALD was assigned as the primary category when alcohol use was judged to be a significant contributing factor.

Cirrhosis was defined as evidence of stage F4 fibrosis on clinical, radiographical, elastographical, or histological assessment, including both compensated and decompensated disease. Non-cirrhosis was defined as CLD without evidence of cirrhosis (F0–F3).

Exclusion criteria included hepatic encephalopathy precluding informed consent, inability to complete surveys in English, or cognitive impairment preventing survey completion. The study was approved by the Institutional Review Board of MGH, and all participants provided informed consent.

### Survey measures

A comprehensive battery of validated and investigator-developed surveys was administered to characterize stigma and related psychosocial constructs relevant to CLD.[20], [21], [22], [23], [24], [25], [26], [27] In addition to the LDSS, we included measures of alcohol use disorder (AUD) stigma, alcohol use severity, mental health symptoms, and attitudes toward AUD and liver disease treatment. These measures were selected to evaluate the convergent validity of the LDSS and to identify psychological and behavioral correlations of stigma.

#### Liver disease stigma scale

Liver disease stigma was measured using the LDSS, which was developed by adapting the validated SU-SMS.^20^ Eighteen SU-SMS items were revised to reference liver disease rather than substance or alcohol use. In addition, 11 new items were created to capture stigma experiences specific to liver disease, particularly AS and ES from healthcare workers and family members. Draft items underwent cognitive interviewing with 20 patients with CLD (16 with ALD and four with other liver disease, 85% of whom had cirrhosis) to assess comprehension and relevance (Supplementary material 1). Items were refined based on patient feedback and reviewed by a multidisciplinary expert panel comprising one hepatologist (WZ), one psychiatrist (AI), one addiction medicine physician (SW), and one social psychologist (AF) to ensure content validity and clinical relevance. One item was revised and one item removed during cognitive interviewing because of redundancy, resulting in a final 28-item refined pool (Table S1).

The LDSS assesses three stigma mechanisms: IS, AS, and ES. The LDSS further subdivides AS and ES into two domains: stigma from family and stigma from healthcare workers. Ultimately, we hypothesized that the LDSS would demonstrate a five-factor structure representing IS, AS from family (AS-FAM), AS from healthcare workers (AS-HC), ES from family (ES-FAM), and ES from healthcare workers(ES-HC). All LDSS items are rated on a 5-point Likert scale (1 = strongly disagree; 5 = strongly agree).

#### AUD stigma

The SU-SMS was also used to assess AUD stigma. The SU-SMS was modified for AUD and contained three six-item subscales measured on 5-point Likert scales (1= very unlikely, 5 = very likely): AS, IS, and ES. Internal consistency reliability was high for all three subscales (α = 0.85, 0.93, and 0.86 for AS, IS, and ES, respectively).

#### Mental health

Depressive symptoms were measured with the two-item Patient Health Questionnaire-2 (PHQ).^23^^,^^24^ Anxiety symptoms were measured with the two-item Generalized Anxiety Disorder Scale-2 (GAD).^21^^,^^23^ Internal consistency reliability was high for both measures (α = 0.84 and 0.87, respectively).

#### Attitudes toward liver disease and alcohol use

Attitudes toward liver disease were measured using seven items adapted from existing attitude and stereotype measures.^8^^,^^10^^,^^25^^,^^26^ Items were designed to assess negative attitudes about people with liver disease (e.g. ‘People with liver disease have only themselves to blame’) and were measured using a 5-point scale (1 = strongly disagree, 5 = strongly agree). Internal consistency was good (α = 0.89). Attitudes toward alcohol use were measured with seven items developed by members of the study team (WZ and ABF). Items were designed to assess the extent to which participants were concerned about their alcohol use (e.g. ‘I am worried that my doctors will be upset with me for drinking alcohol’), Internal consistency reliability was acceptable (α = 0.73). Attitudes toward alcohol use were only assessed in patients with a history of AUD.

#### Treatment-related attitudes

Attitudes toward liver disease treatment were measured with five items developed by the study team and were designed to measure different aspects of treatment avoidance (e.g. ‘I do not deserve to get treatment for my liver disease’). Items were measured on a 5-point scale (1 = strongly disagree, 5 = strongly agree), and internal consistency reliability was good (α = 0.81).

Attitudes toward AUD treatment were measured with a modified version of the Attitudes Toward Treatment subscale of the Endorsed and Anticipated Stigma Inventory.^27^ The subscale contains eight items. The original scale was designed for mental health treatment (e.g. ‘Medications for mental health problems are ineffective); items were modified to reflect AUD treatment (e.g. ‘Medications for alcohol use disorder are ineffective’). Internal consistency reliability for the scale was high (α = 0.87). Attitudes toward AUD treatment were only assessed in patients with a history of AUD.

### Data analysis

Analyses were conducted in R (version 4.3.2; R Foundation for Statistical Computing, Vienna, Austria) and SPSS (version 29.0, IBM Corporation, Armonk, CA, USA). Missing data were minimal (<5% for all variables) and handled using listwise deletion. Consistent with COSMIN guidelines for psychometric evaluation of patient-reported outcome measures, analyses proceeded in sequential steps to evaluate structural validity, internal consistency, construct validity (convergent and known-groups), and item performance.

#### Structural validity (EFA)

EFA using maximum likelihood estimation with oblimin rotation was performed to examine the underlying factor structure of the LDSS. Model selection was guided by eigenvalues, scree plot inspection, factor interpretability, and factor loadings ≥0.40. Items with substantial cross-loadings (>0.40 on two or more factors), loadings on nonhypothesized factors, or factor loadings <0.40 were considered for removal, considering conceptual alignment with the intended subscales. All factor analyses were performed using the original 5-point response scale.

#### Item performance and floor/ceiling effects

For descriptive reporting, response categories collapsed from 5 to 3 points to improve distributional clarity in tables; however, full psychometric testing used the complete 5-point scale. Floor and ceiling effects were examined for each item and subscale, defined as *a priori* as >15% of participants endorsing the lowest or highest response option.

#### Internal consistency

Internal consistency reliability was assessed using Cronbach’s α and McDonald's omega (ω) for each LDSS subscale; α values ≥0.70 were considered acceptable and ≥0.80 preferred.

#### Construct validity

Pearson correlations were used to examine associations between LDSS subscales and theoretically related constructs, including AUD stigma (SU-SMS), mental health symptoms (PHQ-2, GAD-2), attitudes toward alcohol use, and attitudes toward liver disease and liver disease treatment. We expected moderate to strong correlations (r = 0.30–0.70), with the highest associations for IS and mental health symptoms.

Independent sample *t* tests were conducted to determine whether LDSS scores differed between patients with ALD and those with non-ALD liver disease. Consistent with theory and prior literature, we hypothesized that participants with ALD would report higher IS, AS, and ES. For group comparisons, patients with ALD alone or ALD plus another etiology were categorized as ALD; patients without any alcohol involvement were categorized as non-ALD. Additional linear regression models were conducted to examine mean differences between groups while adjusting for age, sex, race, education, mental health history, and cirrhosis status. As an additional known-groups validity analysis, we compared LDSS subscale scores between participants with and without cirrhosis using t tests and linear regression adjusting for age, sex, race, education, mental health history, and ALD status.

## Results

### Patient characteristics

Between August 2023 and August 2024, 277 eligible patients were approached across inpatient and outpatient hepatology services at MGH, of whom 211 (76.2%) provided informed consent and completed the survey. Of the 66 patients (23.8%) who did not participate, 29 declined because of lack of time, 31 because of lack of interest, and six because of lack feeling too unwell or other reasons. The sample included adults with CLD across all etiologies, with 14.2% (n = 30) recruited inpatients and 85.8% (n = 181) recruited outpatients. Participant characteristics are summarized in Table 1. The sample was predominantly White (85.7%), male (55.0%), and had a mean age of 57.3 years (SD = 12.9). Most participants (60.7%, n = 128) had ALD, including those with ALD alone or in combination with other etiologies. Most participants had cirrhosis (73.6%) and at least one non-liver metabolic disease, including hypertension, diabetes, hyperlipidemia, or BMI >25 (75.5%). Common comorbidities included depression (53.6% with any history; mean PHQ-2 score = 1.08, SD = 1.56) and AUD history (52.8%, with 84.5% of those in the ALD group).

**Table 1:** Demographics by ALD status.

Demographics	Total (N = 211)	ALD (n = 128)	Non-alcohol liver disease (n = 83)	*p* value
**Sex**				<0.001
Male	116 (55.2)	85 (66.4)	31 (37.8)	—
Female	94 (44.8)	43 (33.6)	51 (62.2)	—
Gender identity				<0.001
Man	116 (55.0)	84 (65.6)	32 (38.6)	—
Woman	94 (44.5)	43 (33.6)	51 (61.4)	—
Prefer not to answer	1 (0.47)	1 (0.78)	0 (0.00)	—
Race				0.007
White	179 (85.7)	114 (89.1)	65 (80.2)	—
Black	1 (0.48)	1 (0.78)	0 (0.00)	—
Asian	6 (2.87)	2 (1.55)	4 (4.94)	—
Multiracial	12 (5.74)	2 (1.55)	10 (12.3)	—
Native American/Alaskan Native	1 (0.48)	1 (0.78)	0 (0.00)	—
Other	4 (1.91)	3 (2.34)	1 (1.23)	—
Declined to answer	6 (2.87)	5 (3.91)	1 (1.23)	—
Hispanic	12 (5.74)	9 (7.03)	3 (3.70)	0.641
Marital status				0.002
Married	109 (53.7)	56 (45.2)	53 (67.1)	—
Single	58 (28.6)	42 (33.9)	16 (20.3)	—
Divorced	30 (14.8)	24 (19.4)	6 (7.59)	—
Widowed	6 (2.96)	2 (1.61)	4 (5.06)	—
Education				<0.001
Less than high school	7 (3.45)	6 (4.88)	1 (1.25)	—
High school degree	96 (47.3)	71 (57.7)	25 (31.2)	—
Undergraduate degree	66 (32.5)	33 (26.8)	33 (41.2)	—
Graduate degree (*e.g.* MA, MBA)	28 (13.8)	11 (8.94)	17 (21.2)	—
Professional degree (*e.g.* MD, PhD)	6 (2.96)	2 (1.63)	4 (5.00)	—
Employment status				0.213
Yes, full-time	68 (32.4)	35 (27.6)	33 (39.8)	—
Yes, part-time	24 (11.4)	15 (11.8)	9 (10.8)	—
Yes, voluntary work (unpaid)	2 (0.95)	1 (0.79)	1 (1.20)	—
No, currently looking	8 (3.81)	7 (5.51)	1 (1.20)	—
No, not currently looking	108 (51.4)	69 (54.3)	39 (47.0)	—
Personal income (per year)				0.077
No income	21 (10.4)	15 (11.9)	6 (7.89)	—
<US$15,000	21 (10.4)	16 (12.7)	5 (6.58)	—
US$15,000–24,999	24 (11.9)	18 (14.3)	6 (7.89)	—
US$25,000–34,999	11 (5.45)	6 (4.76)	5 (6.58)	—
US$35,000–44,999	13 (6.44)	10 (7.94)	3 (3.95)	—
US$45,000–54,999	11 (5.45)	9 (7.14)	2 (2.63)	—
US$55,000–74,999	26 (12.9)	17 (13.5)	9 (11.8)	—
US$75,000–99,999	19 (9.41)	9 (7.14)	10 (13.2)	—
US$100,000–149,999	30 (14.9)	13 (10.3)	17 (22.4)	—
≥US$150,000	26 (12.9)	13 (10.3)	13 (17.1)	—
Health insurance				0.120
Private health insurance	136 (65.1)	84 (64.8)	53 (65.4)	—
Medicare	59 (28.2)	33 (25.8)	26 (32.1)	—
Medicaid/medical assistance	14 (6.70)	12 (9.38)	2 (2.47)	—
Mental health/alcohol history				
Any history of a mental health problem (% yes)	133 (63.9)	90 (70.9)	43 (53.1)	0.014
Any history of AUD (% yes)	111 (53.1)	108 (84.4)	3 (3.70)	<0.001
Depressive symptoms (PHQ-2)	1.09 (1.56)	1.23 (1.60)	0.87 (1.47)	0.095
Generalized anxiety symptoms (GAD-2)	1.31 (1.75)	1.45 (1.81)	1.11 (1.65)	0.165
AUDIT total	1.85 (3.10)	2.47 (3.70)	0.89 (1.34)	<0.001
CLD etiology				<0.001
Alcohol-associated cirrhosis	60 (28.7)	60 (46.9)	0 (0.00)	—
Alcohol-associated fatty liver	3 (1.44)	3 (2.34)	0 (0.00)	—
Alcohol-associated hepatitis	4 (1.91)	4 (3.12)	0 (0.00)	—
Cholestatic liver disease	19 (9.09)	0 (0.00)	19 (23.5)	—
MASLD	32 (15.3)	0 (0.00)	32 (39.5)	—
Multiple etiologies	77 (36.4)	62 (47.7)	15 (18.5)	—
Viral hepatitis	11 (5.26)	0 (0.00)	4 (4.94)	—
Other	4 (1.91)	0 (0.00)	11 (13.6)	—
CLD stage				<0.001
Non-cirrhosis	55 (26.6)	7 (5.65)	48 (57.8)	—
Cirrhosis	152 (73.4)	117 (94.4)	35 (42.2)	—
Metabolic disease				0.001
No	36 (17.5)	24 (19.2)	12 (14.8)	—
At least 1	63 (30.6)	46 (36.8)	17 (21.0)	—
At least 2	50 (24.3)	33 (26.4)	17 (21.0)	—
At least 3	57 (27.7)	22 (17.6)	35 (43.2)	—
Patient type				0.001
Inpatient	30 (14.2)	27 (21.1)	3 (3.61)	—
Outpatient	181 (85.8)	101 (78.9)	80 (96.4)	—

Data are presented as *n* (%). ALD, alcohol-associated liver disease; AUD, alcohol use disorder; GAD-2, Generalized Anxiety Disorder-2; MASLD, metabolic dysfunction-associated steatotic liver disease; MHP, mental health problem; PHQ-2: Patient Health Questionnaire-2.

Participants with ALD differed from those with non-ALD disease on several characteristics. The ALD group had significantly higher rates of AUD history (84.4% *vs.* 3.7%, *p* <0.001) and cirrhosis (94.4% *vs.* 42.2%, *p* <0.001). The ALD group was also less likely to be married (45.2% *vs.* 67.1%, *p* = 0.003) and have lower educational attainment (*p* <0.001). No significant differences were observed in gender, race, employment status, or health insurance type.

### Structural validity: EFA

The Kaiser–Meyer–Olkin (KMO) measure of sampling adequacy was 0.890, indicating excellent factorability, and Bartlett's test of sphericity was significant (χ^2^ [276] = 4,939.83, *p* <0.001), supporting the appropriateness of factor analysis. The sample-to-item ratio (7.6:1) met established standards for factor analysis.

An EFA using maximum likelihood estimation with oblimin rotation was first conducted on all 28 LDSS items (Table S2). Four items were removed: two did not load on the hypothesized factor, one loaded on multiple factors without a clear primary loading, and one did not reach the prespecified loading threshold (>0.40). One item (‘Healthcare workers assume I engage in risky behavior’) showed cross-loading on both AS-HC (loading = 0.560) and ES-HC (loading = −0.403) subscales. The item was retained in the AS-HC domain based on its stronger primary loading and conceptual alignment. The final LDSS included 24 items: eight assessing IS, nine assessing ES (from both family members and healthcare workers), and seven assessing AS (expectations of stigma from family members and healthcare workers) (Table S3).

The scree plot for the remaining 24 items suggested the extraction of five factors accounting for 73.7% of the total variance. Consistent with our hypothesis based on stigma theory and prior qualitative work, the five factors represented IS, AS-FAM, ES-HC, AS-FAM, and AS-HC. Table 2 presents the final factor structure and loadings for the 24-item LDSS. Factor loadings ranged from 0.50 to 0.99, indicating strong to very strong item–factor relationships.

**Table 2:** Exploratory factor analysis results of the final 24-item LDSS.

Item	Factor				
	IS	ES-FAM	ES-HC	AS-FAM	AS-HC
I think less of myself because I have liver disease	0.990	—	—	—	—
I feel ashamed of having liver disease	0.914	—	—	—	—
I feel I am not as good as others because I have liver disease	0.907	—	—	—	—
Having liver disease makes me feel like I am a bad person	0.895	—	—	—	—
Having liver disease is disgusting to me	0.780	—	—	—	—
I feel useless because I have liver disease	0.719	—	—	—	—
Having liver disease makes me feel mentally weak	0.710	—	—	—	—
I feel I am to blame for having liver disease	0.639	—	—	—	—
Family members have looked down on me	—	0.877	—	—	—
Family members have treated me differently	—	0.831	—	—	—
Family members have thought that I cannot be trusted	—	0.790	—	—	—
Family members have avoided me	—	0.653	—	—	—
Family members assume I have problems with alcohol	—	0.594	—	—	—
Healthcare workers have given me poor care	—	—	-0.908	—	—
Healthcare workers have not listened to my concerns	—	—	-0.790	—	—
Healthcare workers have blamed me for my health problems	—	—	-0.560	—	0.403
Healthcare workers assume I engage in risky behavior	—	—	-0.468	—	—
Family members will avoid me	—	—	—	-0.902	—
Family members will look down on me	—	—	—	-0.877	—
Family members will treat me differently	—	—	—	-0.814	—
Family members will think that I cannot be trusted	—	—	—	-0.501	—
Healthcare workers will assume I have problems with alcohol	—	—	—	—	0.835
Healthcare workers will assume I engage in risky behavior	—	—	—	—	0.775
Healthcare workers will blame me for my health problems	—	—	—	—	0.753

Factor loadings <0.40 have been suppressed. AS-FAM, anticipated stigma from family; AS-HC, anticipated stigma from healthcare workers; ES-FAM, experienced stigma from family; ES-HC, experienced stigma from healthcare workers; IS, internalized stigma.

Internal consistency was excellent for all five subscales: IS (α = 0.97, ω = 0.97, eight items), ES-FAM (α = 0.90, ω = 0.96, five items), ES-HC (α = 0.90, ω = 0.966, four items), AS-FAM (α = 0.92, ω = 0.97, four items), and AS-HC (α = 0.91, ω = 0.95, three items).

### Item performance and floor/ceiling effects

For descriptive presentation only, the five response categories were collapsed into three: strongly disagree/disagree; neutral; and agree/strongly agree (Table 3). IS items showed a broader distribution of responses, with 9.1–12.4% of participants endorsing neutral responses and 12.0–42.1% endorsing agreement across items. The most endorsed item of the IS subscale was ‘I feel I am to blame for having liver disease’, with 42.1% of the sample agreeing or strongly agreeing with the statement. This domain demonstrated relatively limited floor effects, consistent with greater variability in IS within the sample.

**Table 3:** Item- and subscale descriptive statistics for the final LDSS.

Stigma	Mean (SD)	α	ω	Strongly disagree/disagree, n (%)	Neutral, n (%)	Agree/strongly agree, n (%)
Internalized liver disease stigma	2.01 (1.09)	0.97	0.97	—	—	
Having liver disease makes me feel like I am a bad person	—	—	—	156 (75.0)	26 (12.5)	26 (12.5)
I feel I am not as good as others because I have liver disease	—	—	—	153 (73.2)	24 (11.5)	32 (15.4)
I feel ashamed of having liver disease	—	—	—	141 (68.1)	22 (10.6)	44 (21.3)
I think less of myself because I have liver disease	—	—	—	149 (71.6)	21 (10.1)	38 (18.3)
Having liver disease makes me feel mentally weak	—	—	—	155 (74.5)	26 (12.5)	27 (13.0)
Having liver disease is disgusting to me	—	—	—	158 (76.0)	19 (9.13)	31 (14.9)
I feel I am to blame for having liver disease	—	—	—	99 (47.6)	21 (10.1)	88 (42.3)
I feel useless because I have liver disease	—	—	—	167 (80.3)	16 (7.69)	25 (12.0)

				**Never/**N**ot often, n (%)**	**Somewhat often, n (%)**	**Often/very often, n (%)**

Experienced liver disease stigma: family	1.55 (0.92)	0.90	0.96	—	—	—
Family members have thought that I cannot be trusted	—	—	—	185 (86.9)	8 (3.76)	20 (9.39)
Family members have looked down on me	—	—	—	189 (88.7)	10 (4.69)	14 (6.57)
Family members have treated me differently	—	—	—	184 (86.4)	10 (4.69)	19 (8.92)
Family members have avoided me	—	—	—	196 (92.0)	4 (1.88)	13 (6.10)
Family members assume I have problems with alcohol	—	—	—	155 (73.1)	19 (9.00)	37 (17.5)
Experienced liver disease stigma: healthcare workers	1.44 (0.84)	0.92	0.96	—	—	
Healthcare workers have given me poor care	—	—	—	197 (92.5)	7 (3.30)	13 (6.13)
Healthcare workers have not listened to my concerns	—	—	—	192 (90.6)	7 (3.30)	13 (6.13)
Healthcare workers have blamed me for my health problems	—	—	—	192 (90.6)	4 (1.89)	16 (7.55)
Healthcare workers assume I engage in risky behavior	—	—	—	177 (83.5)	19 (8.96)	16 (7.55)

				**Very unlikely/unlikely, n (%)**	**Neither likely/unlikely, n (%)**	**Likely/very likely, n (%)**

Anticipated liver disease stigma: family	1.33 (0.69)	0.92	0.97	—	—	—
Family members will think that I cannot be trusted	—	—	—	192 (92.3)	10 (4.81)	6 (2.88)
Family members will look down on me	—	—	—	193 (91.9)	12 (5.71)	5 (2.38)
Family members will treat me differently	—	—	—	186 (88.6)	14 (6.67)	10 (4.76)
Family members will avoid me	—	—	—	191 (91.4)	14 (6.70)	4 (1.91)
Anticipated liver disease stigma: healthcare workers	1.57 (0.92)	0.91	0.95	—	—	
Healthcare workers will blame me for my health problems	—	—	—	185 (88.1)	15 (7.14)	10 (4.76)
Healthcare workers will assume I have problems with alcohol	—	—	—	168 (80.4)	19 (9.09)	22 (10.5)
Healthcare workers will assume I engage in risky behavior	—	—	—	167 (81.9)	21 (10.3)	16 (7.84)

Cronbach's α and McDonald's ω based on polychoric correlations.

By contrast, AS and ES items showed more pronounced floor effects, with 73.6–92.5% of participants selecting the lowest response category for several items. However, endorsement of stigma (agree/strongly agree) remained present for 2.4–17.5% of participants, depending on the item, indicating that these domains still captured meaningful variability despite lower overall prevalence.

Ceiling effects were minimal across all items, with <10% of participants endorsing the highest response category (Table 3).

### Construct validity

#### Convergent validity

To evaluate construct validity, we examined convergent validity by correlating LDSS subscales with theoretically related measures. Given that the LDSS was adapted from the SU-SMS, we expected moderate to strong correlations with the SU-SMS, reflecting shared underlying stigma mechanisms, while maintaining sufficient distinction to demonstrate liver disease-specific measurement. We also examined correlations with attitude measures and mental health symptoms to confirm that the LDSS assesses stigma as a related but distinct construct.

Table 4 presents correlations between LDSS subscales and related constructs among participants with AUD history (n = 112). LDSS subscales were moderately intercorrelated (r = 0.31–0.57), indicating that they assess related but distinct stigma mechanisms.

**Table 4:** Correlation matrix for liver disease stigma with AUD stigma, treatment intentions and attitudes, and mental health symptoms.

Variable	1	2	3	4	5	6	7	8	9	10	11	12	13
1. LD ES-HC	—	—	—	—	—	—	—	—	—	—	—		
2. LD ES-FAM	0.56∗∗	—	—	—	—	—	—	—	—	—	—		
3. LD AS-HC	0.59∗∗	0.31∗∗	—	—	—	—	—	—	—	—	—		
4. LD AS-FAM	0.38∗∗	0.57∗∗	0.48∗∗	—	—	—	—	—	—	—	—		
5. LD IS	0.32∗∗	0.51∗∗	0.36∗∗	0.42∗∗	—	—	—	—	—	—	—		
6. LD Tx Att	0.39∗∗	0.30∗∗	0.46∗∗	0.53∗∗	0.37∗∗	—	—	—	—	—	—		
7. LD Att	0.33∗∗	0.55∗∗	0.36∗∗	0.47∗∗	0.80∗∗	0.38∗∗	—	—	—	—	—		
8. ALC ES	0.69∗∗	0.80∗∗	0.43∗∗	0.60∗∗	0.40∗∗	0.41∗∗	0.40∗∗	—	—	—	—		
9. ALC AS	0.66∗∗	0.65∗∗	0.55∗∗	0.60∗∗	0.30∗	0.44∗∗	0.33∗∗	0.76∗∗	—	—	—		
10. ALC IS	0.26∗	0.70∗∗	0.19	0.39∗∗	0.53∗∗	0.17	0.52∗∗	0.49∗∗	0.45∗∗	—	—		
11. ALC Tx Att	0.23∗	-00.02	0.40∗∗	0.13	-00.01	0.30∗	0.12	0.07	0.20	0.02	—		
12. ALC Att	0.19	0.42∗∗	0.26∗	0.24∗	0.35∗∗	0.19	0.37∗∗	0.38∗∗	0.22	0.40∗∗	0.31∗∗		
13. PHQ	0.31∗∗	0.37∗∗	0.26∗	0.30∗∗	0.60∗∗	0.37∗∗	0.52∗∗	0.22	0.31∗∗	0.45∗∗	0.11	0.18	
14. GAD	0.29∗∗	0.38∗∗	0.18	0.25∗	0.51∗∗	0.33∗∗	0.42∗∗	0.26∗	0.31∗∗	0.42∗∗	0.06	0.16	0.83∗∗

Subscales of the LDSS were moderately correlated with one another (r = 0.31–0.59), indicating that they measure related but distinct constructs. LDSS subscales were also moderately correlated with attitudes toward liver disease and its treatment (average r = 0.44), except for a large correlation between treatment attitudes and internalized stigma (r = 0.80). Correlations with SU-SMS alcohol stigma subscales ranged from r = 0.19 to 0.80 (average r = 0.52). The strongest association (r = 0.80) occurred between the two experienced stigma subscales. Depression and anxiety symptoms were moderately correlated with all LDSS subscales (r = 0.18–0.60), with the highest correlations observed for internalized stigma (0.60 for depression, 0.51 for anxiety); ∗*p* <0.05. ∗∗*p* <0.01. ALC, alcohol; AS, anticipated stigma; AS-FAM, anticipated stigma from family; AS-HC, anticipated stigma from healthcare workers; Att, attitudes; ES, experience stigma; ES-FAM, experienced stigma from family; ES-HC, experienced stigma from healthcare workers; GAD, Generalized Anxiety Disorder Scale-2; IS, internalized stigma; LD, liver disease; PHQ, Patient Health Questionnaire-2; Tx, treatment.

The LDSS demonstrated strong convergent validity with corresponding SU-SMS alcohol stigma subscales. The correlation between IS subscales (r = 0.53) supports convergent validity with the established SU-SMS while maintaining conceptual distinction. LDSS ES and AS subscales also showed strong correlations with their SU-SMS counterparts (r = 0.43–0.80). As expected, these correlations were robust but not redundant, suggesting that, although the LDSS aligns with established stigma mechanisms, it also captures liver disease-specific experiences distinct from substance use stigma.

LDSS subscales also demonstrated convergent validity with mental health symptoms: all LDSS subscales were positively associated with depressive and anxiety symptoms (r = 0.18–0.60, *p* <0.01), indicating that greater stigma was linked to higher psychological distress (Table 4). IS demonstrated the strongest correlations with both depression (r = 0.60) and anxiety (r = 0.51).

LDSS subscales were also moderately correlated with negative attitudes toward liver disease treatment (r = 0.30–0.53), suggesting that higher stigma, particularly IS, contributes to treatment avoidance or disengagement from care. These findings highlight the clinical importance of addressing stigma as a modifiable barrier to psychological wellbeing and treatment engagement among patients with CLD.

#### Known-groups validity

**Fig. 1:**
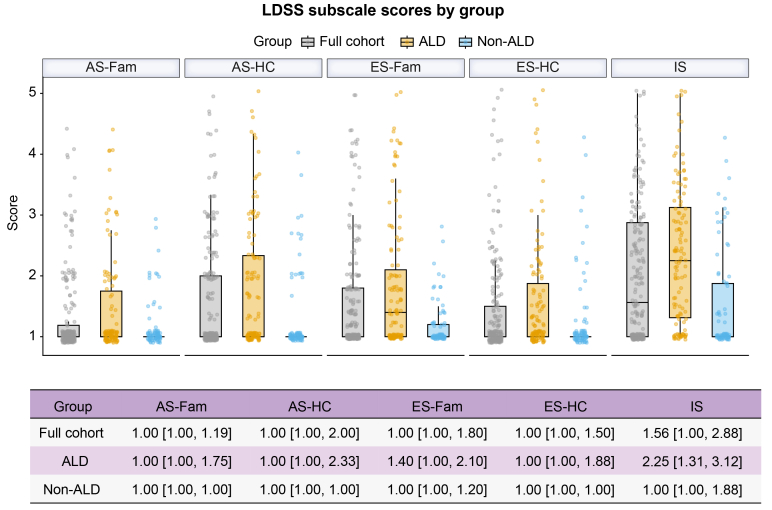
Distributional characteristics (median and IQR) of LDSS subscales by ALD status. Bottom panel contains the median (IQR) for each subscale. AS-Fam, anticipated stigma from family; AS-HC, anticipated stigma from healthcare workers; ES-Fam, experienced stigma from family; ES-HC, experienced stigma from healthcare workers; IQR, interquartile range; IS, internalized stigma.

To evaluate known-groups validity, we compared LDSS scores between participants with ALD and those with non-ALD liver disease. For patients with more than one etiology, ALD was assigned as the primary category when alcohol use was judged to be a significant contributing factor to liver injury. Thus, patients with mixed etiologies involving alcohol were categorized under ALD. Table 5 presents unadjusted and adjusted mean comparisons of individuals with ALD *vs.* those without ALD. In unadjusted analyses, participants with ALD reported significantly higher stigma across all domains (all *p* <0.01), with large effect sizes for IS (d = 0.85) and ES-FAM (d = 0.77) (Fig. 1). After adjusting for age, sex, race, education, mental health history, and cirrhosis status, differences in IS (*p* <0.001) and ES-FAM (*p* <0.001) remained highly significant, with moderate-to-large effect sizes (d = 0.57 and d = 0.50, respectively). ES-total and AS-HC also remained significant, whereas differences in other domains were no longer significant.

**Table 5:** Known-groups validity (LDSS comparisons by type of liver disease).

Group	Unadjusted			Adjusted			Cohen’s d	Cohen’s d 95% CI
	ALD	Non-ALD	*p*	ALD	Non-ALD	*p* (adjusted)		
	Mean (SD)	Mean (SD)		Mean (SE)	Mean (SE)			
Internalized stigma	2.34 (1.12)	1.50 (0.84)	<0.001	2.24 (0.12)	1.70 (0.13)	<0.001	0.57	0.24–0.91
Experienced stigma-total	1.70 (0.90)	1.22 (0.47)	<0.001	1.57 (0.09)	1.29 (0.09)	0.019	0.40	0.07–0.73
Experienced stigma-healthcare	1.58 (0.94)	1.27 (0.67)	0.006	1.45 (0.11)	1.33 (0.11)	0.37	0.15	-0.18 to 0.48
Experienced stigma-family	1.80 (1.08)	1.18 (0.37)	<0.001	1.66 (0.11)	1.25 (0.11)	0.003	0.50	0.17–0.83
Anticipated stigma-total	1.57 (0.79)	1.23 (0.49)	<0.001	1.47 (0.08)	1.30 (0.09)	0.119	0.26	-0.07 to 0.60
Anticipated stigma-healthcare	1.77 (1.03)	1.31 (0.67)	<0.001	1.61 (0.12)	1.37 (0.12)	0.037	0.27	-0.07 to 0.60
Anticipated stigma-family	1.43 (0.81)	1.17 (0.42)	0.003	1.38 (0.08)	1.25 (0.09)	0.228	0.20	-0.13 to 0.54

Unadjusted *p* values based on independent sample t tests; adjusted *p* values based on a linear regression model controlling for age, gender, race, education, mental health history, and cirrhosis status. Adjusted means, SEs, Cohen’s d, and 95% CI were obtained using the emmeans package.^28^ Cohen’s d and 95% CI were calculated for the model-adjusted means. ALD, alcohol-associated liver disease.

As an additional known-groups analysis, we compared LDSS scores between participants with and without cirrhosis. Table 6 presents unadjusted and adjusted comparisons. In unadjusted analyses, participants with cirrhosis reported higher stigma across IS and ES domains. After adjustment for age, sex, race, education, mental health history, and liver disease etiology (ALD *vs.* non-ALD), differences remained significant for IS (*p* = 0.003, d = 0.48), ES-total (*p* = 0.026, d = 0.36), and ES-HC (*p* = 0.019, d = 0.38), whereas differences in AS domains were no longer significant.

**Table 6:** Known-groups validity (mean comparisons by cirrhosis status).

Group	Unadjusted			Adjusted			Cohen’s d	Cohen’s d 95% CI
	No cirrhosis	Cirrhosis	*p* value	No cirrhosis	Cirrhosis	*p* value		
	Mean (SD)	Mean (SD)		Mean (SE)	Mean (SE)			
Internalized stigma	1.79 (1.07)	2.21 (1.07)	0.005	1.74 (0.12)	2.20 (0.13)	0.003	0.48	0.16–0.80
Experienced stigma-total	1.38 (0.62)	1.61 (0.87)	0.026	1.30 (0.09)	1.55 (0.09)	0.026	0.36	0.04–0.68
Experienced stigma-healthcare	1.30 (0.70)	1.56 (0.92)	0.022	1.24 (0.10)	1.55 (0.11)	0.019	0.38	0.06–0.70
Experienced stigma-family	1.44 (0.77)	1.65 (1.01)	0.086	1.35 (0.10)	1.56 (0.11)	0.109	0.26	−0.06 to 0.58
Anticipated stigma-total	1.34 (0.63)	1.51 (0.74)	0.093	1.31 (0.08)	1.47 (0.09)	0.117	0.26	−0.0 to 0.57
Anticipated stigma-healthcare	1.43 (0.86)	1.69 (0.96)	0.042	1.39 (0.11)	1.60 (0.12)	0.133	0.24	−0.08 to 0.56
Anticipated stigma-family	1.28 (0.60)	1.38 (0.76)	0.310	1.25 (0.08)	1.38 (0.09)	0.196	0.21	−0.01 to 0.53

Unadjusted *p* values based on independent sample t tests; adjusted *p* values based on a linear regression model controlling for age, gender, race, education, mental health history, and liver disease etiology. Cohen’s d and 95% CI were calculated for the model-adjusted means.

## Discussion

This study evaluated the psychometric properties of the LDSS, a multidimensional instrument adapted from the SU-SMS to assess IS, AS, and ES among patients with CLD. The measure demonstrated strong psychometric performance across diverse liver disease etiologies and stages, including both ALD and non-ALD populations. Across these samples, EFA supported a clear five-factor structure, and each subscale demonstrated excellent internal consistency. LDSS subscale scores showed strong convergent validity with established measures of alcohol-related stigma and related constructs. These findings support the LDSS as a reliable and theory-informed tool to assess stigma in diverse CLD populations.

Previous research has focused primarily on stigma in viral hepatitis or cirrhosis, with limited investigation into the multidimensional nature of stigma across liver disease etiologies. For example, one study of patients with all-cause cirrhosis found stigma to be prevalent and associated with greater depression, lower social support, and reduced engagement in care, but it did not differentiate between IS, AS, and ES. Similarly, research into HBV-related stigma has often examined single domains rather than the complex mechanisms by which stigma affects behavior and outcomes.^19^ By contrast, the LDSS was developed using established theoretical frameworks and adapted from the validated SU-SMS to capture liver disease-specific features, including family and healthcare-related domains.^20^ The incorporation of both patient and clinician input during item generation further enhances its clinical and ecological validity.

The psychometrically evaluated LDSS fills a crucial gap in stigma measurement for liver disease populations. A key advance is its multidomain assessment, because IS, AS, and ES each represent distinct effects that have been associated in previous research with different emotional and behavioral outcomes. IS has been linked with depression and diminished self-worth; AS can lead to nondisclosure and avoidance of care; and ES has been associated with mistrust, social withdrawal, and disengagement from the healthcare system.[12], [13], [14] Measuring these domains separately allows a more nuanced understanding of how stigma mechanisms and their sources might relate to cognitive, affective, and behavioral outcomes in liver disease. The use of LDSS in hepatology settings might help clinicians recognize patients reporting greater stigma burdens who could benefit from additional psychosocial support.^5^

A key finding of this study was that patients with ALD reported significantly higher stigma across all domains compared with those with non-ALD liver disease, with particularly elevated IS and ES-FAM. These differences persisted after adjustment for age, sex, race, education, mental health history, and cirrhosis status, suggesting that alcohol-related etiology is independently associated with greater perceived stigma beyond differences in disease severity. The effect sizes reveal that patients with ALD experience pronounced differences in IS and ES-FAM, suggesting substantial clinical significance in these domains. By contrast, differences in ES-HC were no longer significant after adjustment, implying that healthcare stigma are more evenly distributed across liver disease etiologies. Stigma scores were also higher among participants with cirrhosis, supporting the sensitivity of the scale to differences in disease severity.

Notably, alcohol attribution items loaded onto distinct stigma domains across relational contexts: ES-FAM and AS-HC, but not ES-HC or AS-FAM. This pattern suggests that attribution-based stigma operates differently depending on the source, with family members and healthcare workers eliciting different stigma concerns. More broadly, healthcare stigma may vary substantially across healthcare settings, reflecting institutional culture, healthcare worker attitudes, and regional differences in alcohol-related care. Future multicenter studies will be important to determine whether healthcare-related stigma differs across clinical settings and systems of care. These findings have important implications for patient care and transplant outcomes, particularly in informing interventions aimed at reducing stigma and improving engagement in care.

### Clinical applications and implications

The LDSS offers several potential applications for hepatology practice and research. As a standardized self-report measure, it could help clinicians identify patients with elevated stigma and tailor clinical interactions accordingly by proactively addressing perceived barriers to care, facilitating referrals to behavioral health or peer support services, or increasing follow-up for patients at risk of disengagement. Given that the LDSS subscales capture distinct stigma mechanisms, the instrument could be integrated into clinical workflows at key decision points, such as initial hepatology consultation, pretransplant psychosocial evaluation, or transitions across care settings.

Beyond individual patient care, the LDSS provides a systematic approach to measuring stigma that might inform discussions of transplant equity, where stigma is a plausible but rarely measured contributor to referral patterns and listing decisions, particularly for patients with ALD. Finally, the LDSS could serve as a psychometrically supported outcome measure in future trials of stigma-reduction strategies, including integrated hepatology-addiction care models, peer support interventions, and healthcare worker education initiatives. Future work is needed to establish clinically meaningful cutoffs and to evaluate whether routine stigma assessment is associated with improved care engagement and patient outcomes.

### Strength and limitations

This study has several strengths, including theory-informed item development, rigorous psychometric evaluation, and inclusion of participants spanning multiple liver disease etiologies. Additional strengths include a relatively high participation rate (76.2%) and the use of multiple complementary approaches to evaluate construct validity.

Several limitations should be acknowledged. The LDSS was developed by adapting items from the SU-SM and refining them through expert review, rather than through formal qualitative concept elicitation with patients with liver disease. Although the LDSS is intended to capture stigma mechanisms that are shared across etiologies, condition specific experiences, such as weight-related judgment in MASLD or contagion-related concerns in viral hepatitis, might not be fully represented and might require supplemental modules in future work.

The sample was recruited from a single tertiary academic medical center and was predominantly White and English-speaking, with over-representation of ALD and cirrhosis. The exclusive use of English-language surveys limits generalizability to non-English-speaking populations, who might experience distinct or more severe stigma. Recruitment did not extend to community-based settings, potentially resulting in conservative estimates of stigma burden. The LDSS has not yet been externally validated in independent samples, which limits confidence in the generalizability of the factor structure and score distributions. In addition, the 23.8% nonparticipation rate introduces potential selection bias; patients with higher stigma burden might have been more motivated to participate in a stigma-focused study, which could result in overestimation of stigma prevalence. The sample size, although adequate for the current psychometric analyses, limits statistical power for subgroup analyses and replication in smaller disease-specific subgroups.

In addition, the cross-sectional design precluded evaluation of test-retest reliability, measurement invariance, and responsiveness to intervention, and clinically meaningful score thresholds have not yet been established. Nonetheless, the observed factor structure, excellent internal consistency, and robust convergent and known-groups validity provide a strong psychometric foundation for future refinement and application of the LDSS.

## Concluding remarks and future directions

Future research should evaluate the LDSS longitudinally to determine its predictive value for care engagement, relapse risk, and transplant eligibility. Cross-validation in larger and more diverse samples and testing responsiveness to stigma-reduction interventions will further support its clinical utility. Applying the LDSS across diverse clinical settings and liver disease populations, including community-based samples and under-represented etiologies, will be essential for advancing equitable, patient-centered care in hepatology.

In conclusion, the LDSS is the first multidimensional scale specifically designed and psychometrically evaluated to measure liver disease-related stigma. Widespread adoption and continued refinement of this tool may improve understanding of the role of stigma in care disparities and guide interventions to promote engagement, recovery, and equity among patients with CLD.

## Abbreviations

ALC, alcohol; ALD, alcohol-associated liver disease; AS-FAM, anticipated stigma from family; AS-HC, anticipated stigma from healthcare providers; AS, anticipated stigma; Att, attitudes; AUD, alcohol use disorder; CLD, chronic liver disease; EFA, exploratory factor analysis; ES-FAM, experienced stigma from family; ES-HC, experienced stigma from healthcare workers; ES, experience stigma; GAD, Generalized Anxiety Disorder Scale-2; KMO, Kaiser–Meyer–Olkin; IS, internalized stigma; LD, liver disease; LDSS, Liver Disease Stigma Scale; MASLD, metabolic dysfunction-associated steatotic liver disease; MGH, Massachusetts General Hospital; MHP, mental health problem; PHQ, Patient Health Questionnaire-2; SU-SM, Substance Use Stigma Mechanisms Scale; Tx, treatment.

## Authors’ contributions

Conceptualization, methodology, investigation, data curation, visualization, writing – original draft, project administration, supervision: WZ. Investigation, data curation: EW. Investigation, writing – review and editing: KH, AI. Resources, writing – review and editing: ES, HY, EB. Investigation, resources, writing – review and editing: SW. Writing – review and editing: TK, CB, RG, JL, RW, RTC, NU. Conceptualization, methodology, validation, formal analysis, visualization, writing –review and editing, supervision: ABF; CS: Investigation, data curation.

## Data availability

Deidentified data and analytic code are available from the corresponding author upon reasonable request. All survey items and scoring materials are included in the supplementary materials.

## Financial support

WZ is supported, in part, by the GI Innovation Award from the Massachusetts General Hospital Division of Gastroenterology. No external funding was used for this study.

## Conflicts of interest

The authors declare no conflicts of interest.

Please refer to the accompanying ICMJE disclosure forms for further details.
