# Supplementary material for: Development and psychometric evaluation of the Liver Disease Stigma Scale (LDSS)
Source: JHEP Rep. 2026 May 6;8(8):101870. doi: 10.1016/j.jhepr.2026.101870 (PMC13352063; doi:10.1016/j.jhepr.2026.101870)
Supplement: Multimedia component 1 [file mmc1.pdf]

Development and psychometric evaluation of the Liver Disease Stigma Scale (LDSS)

Wei Zhang, Edward Wu, Hsu Kelly, Cassidy Sun, Toshali Katyal, Cristal Brown, Ana Ivkovic, Heidi Yeh, Emily Bethea, Sarah Wakeman, Russell Goodman, Jay Luther, Robert Wong, Esperance Schaefer, Raymond T Chung, Nneka Ufere, Annie B Fox

Table of contents

Supplement 1…………………………………………………………...........2

Supplement 2…………………………………………………………...........3

Supplement 3…………………………………………………………...........5

Supplement 4…………………………………………………………...........7

Supplement 1. Cognitive Interview and Patient Feedback Guide

Cognitive Interview Procedures

Cognitive interviewing was conducted to evaluate the clarity, relevance, and acceptability of draft items in the Liver Disease Stigma Scale (LDSS). This method is commonly used in PROM development and is suitable for clinical populations with limited time or a high symptom burden.

A convenience sample of 20 adults with chronic liver disease reviewed the draft items. Participants were asked to read all items and provide open-ended feedback on the clarity, comprehension, and emotional tone. Feedback was recorded and used to guide revisions.

Cognitive Interview Guide

1. Were any items confusing or unclear?
2. Did any items feel too strong or emotionally difficult?
3. Did any items feel repetitive?

Summary of Patient Feedback

– One item ("I feel flawed and incomplete because of my liver disease") was removed due to redundancy.

– One item ("Having liver disease makes me feel unclean") was revised to "mentally weak" to improve acceptability.

Supplement 2 Original 29-item LDSS and SU-SMS

#	LDSS Item (initial pool)	SU-SMS Source	Type	Status after cognitive interview
**Internalized Stigma Items**				
1	Having liver disease makes me feel like I'm a bad person.	Having used alcohol and/or drugs makes me feel like I'm a bad person.	Condition adapted (substance use → liver disease)	Retained
2	I feel I'm not as good as others because I have liver disease.	I feel I'm not as good as others because I used alcohol and/or drugs.	Condition adapted	Retained
3	I feel ashamed of having liver disease.	I feel ashamed of having used alcohol and/or drugs.	Condition adapted	Retained
4	I think less of myself because I have liver disease.	I think less of myself because I used alcohol and/or drugs.	Condition adapted	Retained
5	Having liver disease makes me feel mentally weak.	Having used alcohol and/or drugs makes me feel unclean.	**Revised for tone ("unclean" → "mentally weak"),** condition adapted	**Retained (Revised)**
6	Having liver disease is disgusting to me.	Having used alcohol and/or drugs is disgusting to me.	Condition adapted	Retained
7	I feel I am to blame for having liver disease.	None	**New item (LD specific)**	Retained
8	I feel useless because I have liver disease.	None	**New item (LD specific)**	Retained
9	I feel flawed and incomplete because of my liver disease.	None	**New item (LD specific)**	**Removed (redundant)**
**Experienced Stigma Items**				
**10**	Family members have thought that I cannot be trusted.	Family members have thought that I cannot be trusted.	Identical wording	Retained
**11**	Family members have looked down on me.	Family members have looked down on me.	Identical wording	Retained
12	Family members have treated me differently.	Family members have treated me differently.	Identical wording	Retained
13	Family members have avoided me.	None	**New item (LD specific)**	Retained
14	Family members assume I have problems with alcohol.	None	**New item (LD specific)**	Retained
15	Healthcare workers have not listened to my concerns.	Healthcare workers have not listened to my concerns.	Identical wording	Retained
16	Healthcare workers have blamed me for my health problems.	Healthcare workers have thought that I'm pill shopping or trying to con them into giving me prescription medications to get high or sell.	Condition adapted	Retained
17	Healthcare workers have given me poor care.	Healthcare workers have given me poor care.	Identical wording	Retained
18	Healthcare workers assume I engage in risky behavior.	None	**New item (LD specific)**	Retained
19	Healthcare workers assume I have problems with alcohol.	None	**New item (LD specific)**	Retained
**Anticipated Stigma Items**				
20	Family members will think that I cannot be trusted.	Family members will think that I cannot be trusted.	Identical wording	Retained
21	Family members will look down on me.	Family members will look down on me.	Identical wording	Retained
22	Family members will treat me differently.	Family members will treat me differently.	Identical wording	Retained
23	Family members will avoid me.	None	**New item (LD specific)**	Retained
24	Family members will assume I have problems with alcohol.	None	**New item (LD specific)**	Retained

25	Healthcare workers will not listen to my concerns.	Healthcare workers will not listen to my concerns.	Identical wording	Retained
26	Healthcare workers will blame me for my health problems.	Healthcare workers will think that I'm pill shopping or trying to con them into giving me prescription medications to get high or sell.	Condition adapted	Retained
27	Healthcare workers will give me poor care.	Healthcare workers will give me poor care.	Identical wording	Retained
28	Healthcare workers will assume I have problems with alcohol.	None	**New item (LD specific)**	Retained
29	Healthcare workers will assume I engage in risky behavior.	None	**New item (LD specific)**	Retained

LD: Liver disease

Supplement 3. Complete Factor Loadings for the 28-Item LDSS (Exploratory Factor Analysis)

Item	IS	ES-FAM	ES-HC	AS-FAM	AS-HC
Having liver disease makes me feel like I'm a bad person.	0.900				
I feel I'm not as good as others because I have liver disease.	0.914				
I feel ashamed of having liver disease.	0.925				
I think less of myself because I have liver disease.	1.003				
Having liver disease makes me feel mentally weak.	0.703				
Having liver disease is disgusting to me.	0.779				
I feel I am to blame for having liver disease.	0.640				
I feel useless because I have liver disease.	0.705				
Family members have thought that I cannot be trusted.		–0.735			
Family members have looked down on me.		–0.829			
Family members have treated me differently.		–0.812			
Family members have avoided me.		–0.676			
Family members assume I have problems with alcohol.		–0.612			
Healthcare workers have not listened to my concerns.			–0.781		
Healthcare workers have blamed me for my health problems.			–0.579		
Healthcare workers have given me poor care.			–0.884		
Healthcare workers assume I engage in risky behavior.			–0.488		
**Healthcare workers assume I have problems with alcohol. †**					0.431
Family members will think that I cannot be trusted.				0.516	
Family members will look down on me.				0.860	

Family members will treat me differently.				0.809	
Family members will avoid me.				0.889	
**Family members will assume I have problems with alcohol. †**				0.410	0.419
**Healthcare workers will not listen to my concerns. †**				0.450	
Healthcare workers will blame me for my health problems.					0.704
**Healthcare workers will give me poor care.†**					
Healthcare workers will assume I have problems with alcohol.					0.817
Healthcare workers will assume I engage in risky behavior.					0.726

Note. Extraction method: Maximum likelihood. Rotation method: Oblimin with Kaiser normalization. Rotation converged in 18 iterations. All loadings are shown. IS = internalized stigma; ES-FAM = experienced stigma from family; ES-HC = experienced stigma from healthcare workers; AS-FAM = anticipated stigma from family; AS-HC = anticipated stigma from healthcare workers.

† Item removed from the final 24-item LDSS.

Supplement 4 Final 24-item LDSS with Scoring Instructions

#	LDSS Item	Subscale
**Internalized Stigma | Scoring: Mean of items 1–8**		
1	Having liver disease makes me feel like I'm a bad person.	IS
2	I feel I'm not as good as others because I have liver disease.	IS
3	I feel ashamed of having liver disease.	IS
4	I think less of myself because I have liver disease.	IS
5	Having liver disease makes me feel mentally weak.	IS
6	Having liver disease is disgusting to me.	IS
7	I feel I am to blame for having liver disease.	IS
8	I feel useless because I have liver disease.	IS
**Experienced Stigma | Scoring: Mean within each subscale**		
**ES-Family (items 1–5)**		
1	Family members have thought that I cannot be trusted.	ES-Fam
2	Family members have looked down on me.	ES-Fam
3	Family members have treated me differently.	ES-Fam
4	Family members have avoided me.	ES-Fam
5	Family members assume I have problems with alcohol.	ES-Fam
**ES-Healthcare (items 6–9)**		
6	Healthcare workers have not listened to my concerns.	ES-HC
7	Healthcare workers have blamed me for my health problems.	ES-HC
8	Healthcare workers have given me poor care.	ES-HC
9	Healthcare workers assume I engage in risky behavior.	ES-HC
**Anticipated Stigma | Scoring: Mean within each subscale**		
**AS-Family (items 1–4)**		
1	Family members will think that I cannot be trusted.	AS-Fam
2	Family members will look down on me.	AS-Fam
3	Family members will treat me differently.	AS-Fam
4	Family members will avoid me.	AS-Fam
**AS-Healthcare (items 5–7)**		
5	Healthcare workers will blame me for my health problems.	AS-HC
6	Healthcare workers will give me poor care.	AS-HC
7	Healthcare workers will assume I have problems with alcohol.	AS-HC

Note. Anchors for all items: 1 = Strongly Disagree, 5 = Strongly Agree

IS = internalized stigma; ES-FAM = experienced stigma from family; ES-HC = experienced stigma from healthcare workers; AS-FAM = anticipated stigma from family; AS-HC = anticipated stigma from healthcare workers
